# The clinical outcomes of high neutralizing antibodies titer convalescent plasma therapy in early developed severe COVID-19 patients; a case–control study

**DOI:** 10.1186/s12941-022-00542-2

**Published:** 2022-11-19

**Authors:** Nuttakant Nontawong, Taweegrit Siripongboonsitti, Kriangkrai Tawinprai, Mana Boonpratoom, Nawin Krailassiri, Chayaporn Boonkhum, Kamonwan Soonklang, Yong Poovorawan, Nithi Mahanonda

**Affiliations:** 1 Department of Medicine, Prachathiput Hospital, Pathum Thani, Thailand; 2 Division of Infectious Diseases, Department of Medicine, Chulabhorn Hospital, Chulabhorn Royal Academy, Bangkok, Thailand; 3 Princess Srisavangavadhana College of Medicine, Chulabhorn Royal Academy, Bangkok, Thailand; 4 Naungsau Hospital, Pathum Thani, Thailand; 5 Center of Learning and Research in Celebration of HRH Princess Chulabhorn 60th Birthday Anniversary, Chulabhorn Royal Academy, Bangkok, Thailand; 6 Center of Excellence in Clinical Virology, Department of Pediatrics, Faculty of Medicine, Chulalongkorn University, Bangkok, Thailand; 7 Chulabhorn Hospital, Chulabhorn Royal Academy, Bangkok, Thailand

**Keywords:** COVID-19, Convalescent plasma, Severe COVID-19, Mortality

## Abstract

**Background:**

Coronavirus disease 2019 (COVID-19) causes life-threatening pneumonia. Convalescent plasma therapy (CPT) is expected to be the effective COVID-19 treatment for passive immunity. The high neutralizing antibodies titer of CPT is needed to prove the benefit in early developed severe COVID-19.

**Objective:**

This case–control study evaluated transfusion efficacy and adverse events with high-titer (≥ 1:320) COVID-19 convalescent plasma compared with standard care alone in severe COVID-19 pneumonia.

**Results:**

Among 107 severe COVID-19 patients, 55 received CPT plus standard care, and 52 received standard care alone. All-cause mortality was 15.3% in the CPT group compared with 85.4% in the standard care group (p < 0.001). Univariate and multivariate analyses revealed reduced mortality with CPT (HR 0.14; 95% CI 0.07–0.31; p < 0.001 and HR 0.26; 95% CI 0.08–0.79; p = 0.018, respectively). CPT resulted in decreased use of mechanical ventilation, duration of supplemental oxygen, and high-flow oxygen requirement. Clinical and radiological outcomes improved.

**Conclusions:**

Immediate high neutralizing antibody titer CPT is safe and reduces mortality in early developed severe COVID-19 patients. The benefit of CPT in the early course of illness is challenging and requires additional study.

*Trial registration* Thai clinical trials registry (TCTR) no. 20220101003.

## Introduction

The coronavirus disease 2019 (COVID-19) rapidly became a pandemic and caused life-threatening pneumonia, especially in patients with comorbidities. Many repurposed drugs were used and expected to cure the epidemic virus. COVID-19 spread rapidly in Thailand, which encountered the daily spreading of 23,418 COVID-19 cases and 312 deaths per day from July 27, 2021, to August 29, 2021 [1]. To date, treatments to cure or prevent COVID-19 are insufficient. The COVID-19 convalescent plasma (CCP) transfusion has been reported to treat emerging and re-emerging infectious diseases such as MERS-CoV, SARS-CoV-1, and influenza virus in the past century [2–7]. COVID-19 convalescent plasma therapy (CPT) acts as passive immunization, and there are many reports of effective treatment of severe COVID-19.

A recent CPT study revealed that almost all patients had clinical improvement 7 days after CCP transfusion [8]. However, a large randomized control trial (RCT) of 11,558 patients failed to improve 28-day mortality with CPT compared with standard care. The PLACID trial revealed that CPT in hospitalized hypoxemic patients had no benefit in decreasing death or preventing deterioration. A limitation of both studies included the low and varying CCP neutralizing antibody titer [9, 10]. The RCTs in severe COVID-19 showed high neutralizing antibodies titer CPT showed a favorable outcome [11]. The study in 20,000 hospitalized US patients demonstrated safety and that CPT could reduce mortality if high-titer CCP were early transfused [12]. The incidence of serious adverse events in severe COVID-19 patients was < 1%. The 7-day mortality rate reported in a recent study was 14.9% [13]. The ongoing studies of CPT included post-exposure prophylaxis and a combination of CPT in severe and critical illnesses [14].

The role of CPT was controversial in severe illnesses, and some were concerned about antibody-dependent enhancement (ADE) [15]. The barrier to using CPT in hospitalized patients was the need for assays to measure high neutralizing antibodies titer CCP, which were not widely available during the pandemic. Nevertheless, high-titer CPT might offer a compelling treatment option, particularly in resource-limited countries with a shortage of drugs and resources, providing possibilities for treating new variants of concern [16, 17]. Our study aims to evaluate the efficacy of early high neutralizing antibody titer CPT for severe COVID-19 and the effect on mortality.

## Material and methods

This retrospective case–control study revealed CPT in participants with severe COVID-19 at Prachathiput hospital, Pathum Thani province, Thailand, from June 1 to September 30, 2021. The data of the patients were reviewed from medical records. The study protocol was registered in the Thai clinical trials registry (TCTR) no. 20220101003 and was ethically reviewed and approved by the Chulabhorn Ethics Committee no. 205/2564.

**Fig. 1:**
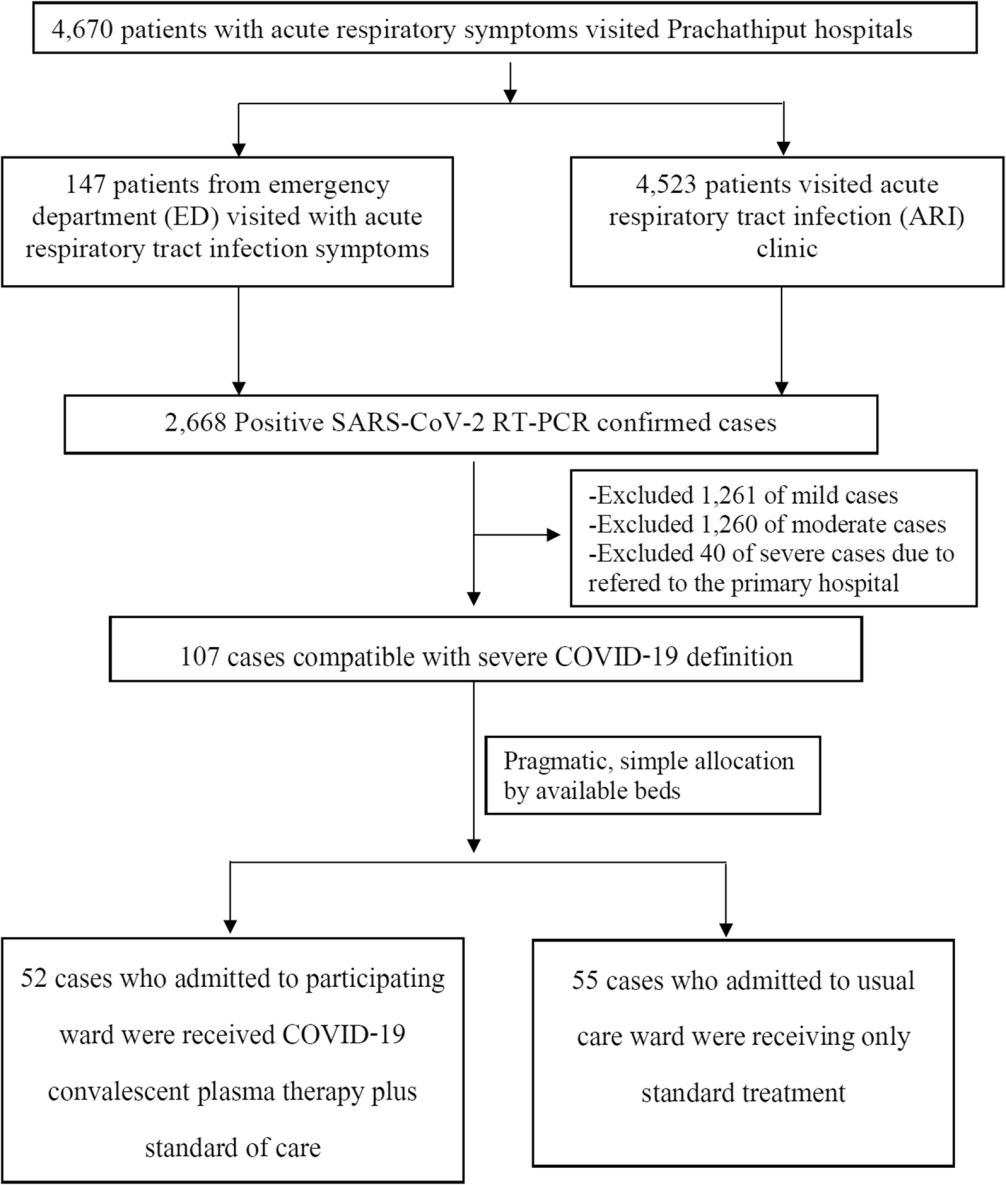
Flow chart of severe COVID-19 enrollment and allocation

Among 2668 RT-PCR confirmed SARS-CoV-2 infected cases, 107 severe COVID-19 patients were hospitalized and allocated by pragmatic, simple allocation to participating or usual care ward depending on available beds. The 55 patients were admitted to the participating ward and received adjunctive CPT to standard therapy, and 52 were admitted to the usual cohort ward and received standard treatment alone (Fig. 1).

### COVID-19 convalescent plasma preparation

Convalescent-plasma-specific donors were selected from a 28-day recent SARS-CoV-2 PCR-confirmed infection. More than 50 kg of male donors were selected to complete the set amount of plasma volume ≥ 250 ml and no possibility of pregnancy, aged 18–60 years who recovered from respiratory tract infection symptoms and meet the criteria for plasma donation. A Micro-neutralization assay was conducted at the faculty of sciences, Mahidol University, to assess neutralizing antibody titer. All recruited donors had neutralizing antibody titer ≥ 1:320, which is above the ≥ 1:160 Food and Drug Administration recommended that was correlated with anti-spike-RBD ELISA IgG titer is ≥ 1:1350 U/ml, which acceptable CCP with high titers of anti-SARS-CoV-2 Antibodies [11, 18]. The eligibility of suitable donors was an assessment of the donor’s medical history in the past 12 months, physical examination relevant to transfusion-transmitted infection risk, and the interval of donating plasma not less than 8 weeks by plasmapheresis if the donor has donated a unit of whole blood or a single unit of red blood cells by apheresis. The human leukocyte antigen antibodies screening was done and interpreted with negative results. The CCP collection was performed by plasmapheresis and processed at the national blood center, Thai Red Cross Society. All CCP was screened for transfusion-transmitted infection, including HIV, hepatitis B and C, syphilis, and malaria, and was processed for pathogen inactivation and stored at− 20 °C for < 1 year.

### Convalescent plasma therapy

The CCP with an ABO-type compatible blood group was transfused for the patients. The 250 and 300 ml of COVID-19 convalescent plasma were transfused to < 60 kg bodyweight recipients and ≥ 60 kg, respectively. The infusion rate was within 2 h, and the rate adjustment depended on the patient’s status and risk for volume overload. During the transfusion until 1 h post CPT, the transfusion-related reaction and adverse events such as fever, chill, rash, anaphylaxis shock, or others were closely monitored and promptly treated.

### Standard treatment

The standard treatment was based on Thailand's national COVID-19 treatment guidelines [19]. The key recommendations were to use 5–10 days of 3600 mg/day of favipiravir (an RNA-dependent RNA polymerase inhibitor) on day 1. This is followed by favipiravir 1600 mg on days 2–10 in severe COVID-19 combined with 5 days of lopinavir 800 mg and ritonavir 200 mg with/without dexamethasone 6–20 mg/day and symptomatic treatment. In obese patients, the loading dose 4800 mg/day of favipiravir on day 1 followed by 2000 mg on days 2–10.

### Criteria for CPT

The severe COVID-19 patients were defined as those who were > 17-year-old, PCR-confirmed COVID-19 pneumonia with significant hypoxemia (oxygen saturation (SpO2) < 94%). The severe COVID-19 patients were admitted to participating wards or the usual care ward during the study period according to bed availability. CPT was initiated as soon as the patient with severe COVID-19 was admitted to the participating ward within 2 days. The repeated second dose of CCP was transfused within 1–3 days after the first dose, followed by an evaluation of the patient’s World Health Organization (WHO) clinical progression scale (increase ≥ 2 from baseline).

### Main outcomes and measures

The primary objective was the 14-day all-cause mortality from severe COVID-19 patients in the hazard ratio (HR). The other objective was oxygen supplement, the adverse events or transfusion reaction, and the WHO clinical progression scale.

### Patient consent statement

All severe COVID-19 patients were informed about the treatment, and all patients had written the consent forms before admission to receive all treatment options that might benefit all severe COVID-19 patients in addition to a standard of care during the outbreak. No specific convalescent plasma consent was required and approved by the Ethics Committee for Human Research, Chulabhorn Research Institute (EC No.205/2564).

### Statistical analysis

Descriptive statistics in frequency and proportion were used in categorical variables. The continuous variables were analyzed and presented in median and interquartile ranges (IQR). The Mann–Whitney U test was used to compare continuous variables. Pearson χ2 and Fisher’s exact tests were used to comparing discrete variables. Time-to-event data were demonstrated using the Kaplan–Meier curve. The difference between Kaplan–Meier curves was tested with the log-rank test. HRs were calculated using Cox proportional hazard regression for the primary endpoint of the HR. The univariate and multivariate analyzed the contributing factors for death. Statistical analyses were performed with STATA/SE 16.1. Statistical significance was defined using a 2-sided significance level of α = 0.05.

## Results

### Clinical outcomes

Among 107 severe COVID-19 patients, 52 (48.5%) received CPT, and 55 (51.4%) received standard care. The median age was 61 and 77 years in the CPT and standard care groups, respectively (p < 0.001). Comorbidities were present in 82.69% of the CPT group and 69.23% of the standard care group (p = 0.108). The most common comorbidities in both groups were hypertension, diabetes, obesity, and chronic kidney disease. There was no difference in duration from the onset of symptoms to admission (5.5 days versus 4 days in the CPT and standard care group, respectively; p = 0.067). However, initial oxygen saturation was lower in the CPT group than in the standard care group (p = 0.007). There was no difference in duration of < 20 mg dexamethasone treatment between groups (p = 0.507). Duration of RdRp inhibitor treatment from the onset of symptoms was longer in the CPT group compared with standard care (10 days [IQR 5–10] and 5.5 days [IQR 1–10], respectively; p = 0.016). Duration from the admission day to critical illness in the CPT group was 5 days, which was longer than the standard care group, which was almost all critical illness on the admission date (p < 0.001) (Table 1).

**Table 1:** Baseline Characteristics

Variable	CPT [n = 52; n (%)]	Standard Care [n = 55; n (%)]	P-value
Sex			0.482^a^
Male	22 (42.31)	27 (49.09)	
Female	30 (57.69)	28 (50.91)	
Age in years, median (IQR)	61 (45–66.5)	71 (61–77)	< 0.001^b^
BMI in kg/m^2^, median (IQR)	26.67 (23.53–31.63)	25.63 (22.04–29.52)	0.257^c^
< 31	24 (60.00)	38 (80.85)	0.106^d^
31–35	10 (25.00)	6 (12.77)	
> 35	6 (15.00)	3 (6.38)	
Comorbidity	43 (82.69)	36 (69.23)	0.108^a^
Hypertension	29 (55.77)	32 (61.54)	0.550^a^
Diabetes Mellitus	25 (48.08)	21 (40.38)	0.430^a^
Cerebrovascular disease	1 (1.92)	1 (1.92)	1.000^d^
Chronic kidney disease	5 (9.62)	10 (19.23)	0.163^a^
End stage renal disease	1 (1.92)	1 (1.92)	1.000^d^
Cancer	1 (1.92)	1 (1.92)	1.000^d^
Ischemic Heart Disease	1 (1.92)	6 (11.54)	0.112^d^
Others	3 (5.76)	2 (3.64)	0.679^b^
Duration from the onset of symptoms to the admission day (days), median (IQR)	5.5 (0–19)	4 (0–16)	0.067^b^
Receiving dexamethasone	52/52 (100%)	55/55 (100%)	1.000
Receiving RdRp inhibitor treatment	52/52 (100%)	55/55 (100%)	1.000
Day of dexamethasone treatment, median (IQR)	8 (0–14)	6 (1–20)	0.507^c^
Day of RdRp inhibitors treatment, median (IQR)	10 (5–10)	5.5 (1–10)	0.016^c^
Day of CPT after the onset of symptoms, median (IQR)	9 (6–13)	0	–
Day of CPT after admission, median (IQR)	2 (1–3)	0	–
The lowest SpO_2_ from admission to deterioration, median (IQR)	91 (68–95)	87 (15–95)	0.007^b^
Duration from the day of admission to critical illness (days), median (IQR)	5 (0–19)	0 (0–13)	< 0.001^b^

^a^Chi-square test

^b^Mann-Whitney U test

^c^Independent t-test

^d^Fisher’s exact test

*BMI* body mass index, *CPT* convalescent plasma therapy, *IQR* interquartile range, *RdRp* RNA-dependent RNA polymerase, *SpO*_*2*_ oxygen saturation

Although the duration from the onset of symptoms to the admission day in the CPT group was no different from the standard care group (5.5 days [IQR 3–8] versus 4 days [IQR 2–7]; p < 0.067), the study's primary outcome indicated 14- and 28-day all-cause mortality of 15.38% in the CPT group, which was significantly lower than 85.45% in the standard care group (P < 0.001). (Table 2).

**Table 2:** Clinical Outcomes of COVID-19 Convalescent Plasma Therapy Versus Standard Care

Variable	CPT (n = 52)	Standard Care (n = 55)	P-value
14-day all-cause mortality, n (%)	8 (15.38)	47 (85.45)	< 0.001^a^
14-day severe COVID-19 death, n (%)	7 (13.46)	47 (85.45)	< 0.001^a^
28-day all-cause mortality, n (%)	8 (15.38)	47 (85.45)	< 0.001^a^
28-day severe COVID-19 death, n (%)	7 (13.46)	47 (85.45)	< 0.001^a^
Duration of supplemental oxygen (days); median (IQR)	5 (4–9)	15 (9.5–18)	< 0.001^b^
Receiving mechanical ventilation, n (%)	44/52 (84.61)	47/55 (85.45)	0.903^a^
Receiving high flow nasal cannula, n (%)	16/52 (30.77)	27/55 (49.08)	0.053^a^
High-flow oxygen therapy; mask with bag, n (%)	11/52 (21.15)	39/55 (70.91)	< 0.001^a^
Low-flow oxygen cannula, n (%)	21/52 (40.38)	21/55 (38.18)	0.816^a^
Length of stay (Days); median (IQR)	10 (3–21)	7 (1–56)	< 0.001^b^
WHO clinical progression scale < 4 on day 3 after admission	1 (1.92)	0 (0)	0.486^c^
WHO clinical progression scale < 4 on day 5 after admission	6 (11.54)	0 (0)	0.027^c^
WHO clinical progression scale < 4 on day 8 after admission	23 (44.23)	0 (0)	< 0.001^a^
WHO clinical progression scale < 4 on day 14 after admission	38 (73.08)	2 (7.27)	< 0.001^a^
Improvement of pneumonia from chest radiography on day 7	26/52 (50)	0/55 (0)	< 0.001^a^
SpO_2_ after weaning of supplemental oxygen n (%)	96 (96–99)	89.5 (73–98)	< 0.001^a^
Number of patients success in weaning oxygen support	44/52 (84.6)	8/55 (14.5)	< 0.001^a^

^a^Chi-square test

^b^Mann-Whitney U test

^c^Fisher’s exact test

*CPT* convalescent plasma therapy, *SpO*_*2*_ oxygen saturation, *IQR* interquartile range, *WHO* World Health Organization

**Fig. 2:**
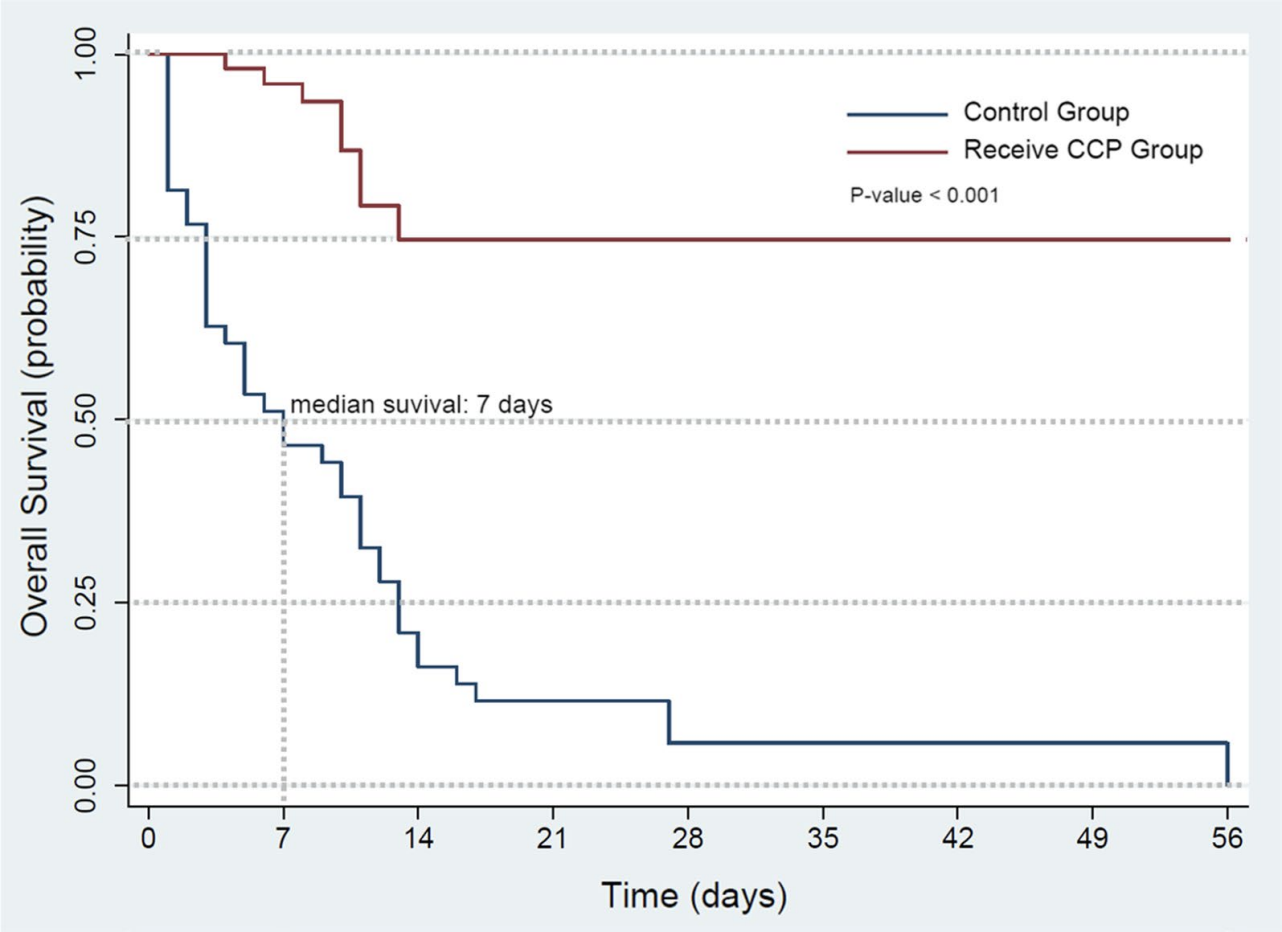
Survival curve of COVID-19 convalescent plasma therapy compared with standard treatment group

In the survival analysis, the median survival time in the standard care group was day 7 after the onset of symptoms. While the survival rate in the CPT group was 85%, statistically significantly higher than the 15% in the standard care group (P < 0.001) on day 14 after admission. (Fig. 2). The length of stay was prolonged in the CPT group than in the standard care group (10 days [IQR 3–21] versus 7 days IQR [1–56]).

The duration of supplemental oxygen was 5 days (IQR 4–9) in the CPT group and 15 days (IQR 9.5–18) in the standard care group (p < 0.001). Successful oxygen weaning was achieved in 84.6% and 14.5% of patients in the CPT and standard care groups, respectively (p < 0.001). There was no difference in mechanical ventilation use between groups—more patients required high-flow oxygen therapy in the standard care group. The oxygen saturation after successful weaning of supplemental oxygen was 96% (IQR 96–99) in the CPT group and 89.5% (IQR 73–98) in the standard care group (p < 0.001).

No WHO clinical progression scale (score < 4) improvement was observed in the standard care group. While there was 11.54% (p = 0.027) and 44.23% in the CPT group, the WHO clinical progression scale was < 4 on day 5 and day 8 (p < 0.001) after admission, respectively. Moreover, the WHO clinical progression scale < 4 on day 14 after admission was 73.08% in the CPT group and 7.27% in the control group (p < 0.001). The CPT group showed more significant improvement in chest X-rays on day 7 of admission than the standard care group. (Table 2).

Cox-regression multivariate analysis revealed that patient age was related to increasing mortality (HR 1.06; 95% confidence interval [CI] 1.02–1.10; p = 0.003), and administration of CPT was related to decreasing mortality (HR 0.26; 95% CI 0.08–0.79; p = 0.018). However, the univariate analysis demonstrated that age, day of starting dexamethasone, favipiravir treatment, and receiving CPT were all associated with mortality with a HR of 1.03 (95% CI 1.01–1.05; p = 0.001), 0.87 (95% CI 0.78–0.97; p = 0.012), 0.79 (95% CI 0.70–0.90; p < 0.001) and 0.14 (95% CI 0.07–0.31; p < 0.001), respectively. (Table 3).

**Table 3:** Cox-regression Univariate and Multivariate Analysis of Factors Affecting Mortality Rate in the CPT Group

Variable	Univariate			Multivariate		
	HR	95%CI	P-value	HR	95%CI	P-value
Sex						
Male	1.21	0.68–2.16	0.508	–	–	–
Female	Ref					
Age	1.03	1.01–1.05	0.001	1.06	1.02–1.10	0.003
BMI (kg/m^2^)	0.99	0.94–1.03	0.497	–	–	–
Underlying disease						
Hypertension	1.22	0.67–2.23	0.506	–	–	–
Diabetes mellitus	0.67	0.36–1.22	0.187	–	–	–
Chronic kidney disease	1.34	0.65–2.80	0.426	–	–	–
Obesity	0.95	0.37–2.41	0.908	–	–	–
Day of starting dexamethasone treatment	0.87	0.78–0.97	0.012	0.66	0.48–0.91	0.11
Day from onset of symptoms to starting favipiravir	0.79	0.70–0.90	< 0.001	1.11	0.80–1.54	0.548
Receiving CPT	0.14	0.07–0.31	< 0.001	0.26	0.08–0.79	0.018

*BMI* body mass index, *CI* confidence interval, *CPT* convalescent plasma therapy, *HR* hazard ratio, *Ref* reference group

### Adverse events

One patient in the CPT group had a transfusion-related reaction. Maculopapular rash occurred within 1 h after CCP transfusion. The rash fully resolved after stopping the CCP transfusion. No serious adverse events were observed in the study.

## Discussion

The real-world observational study demonstrated the advantages of high-neutralizing antibody titer (> 1:320) convalescent plasma therapy for severe COVID-19. Compared with standard care, there was lower all-cause mortality (15%) in the CPT arm with a low HR by univariate and multivariate analysis. Survival analysis showed that CPT resulted in a significantly higher survival rate than standard care. The median survival time with standard care was 7 days after admission. Nevertheless, the standard of care group tended to have lower oxygen saturation on admission and more elderly patients than the CPT-treated group.

Our study demonstrated that high titer (> 1:320) CPT reduced 14- and 28-day mortality from severe COVID-19, reducing the duration of supplemental oxygen and decreasing high-flow oxygen requirements. The CPT group improved the WHO clinical progression scale on day 5 and the radiologic parameter on day 7. A recent study showed high-titer (1:80–1:320) CCP transfused within 3 days of COVID-19 diagnosis related to decreasing mechanical ventilation and mortality [2, 20, 21]. Similarly, an RCT showed a 57% reduction in mortality rate with high-titer (> 1:160) CPT (13%) compared with control (25%) (odds ratio 0.43; p < 0.001) [16]. Our findings correlated with the recent study that demonstrated the relationship between reduced mortality and early transfusion time, and high antibody CPT provided favorable efficacy in hospitalized patients’ treatment [20, 21].

In contrast, the CONCOR-1 study did not show a benefit of CCP in reducing mortality, intensive care unit admission, length of stay, or risk of endotracheal intubation. The RECAP-MAP study did not demonstrate that CPT was associated with a difference in mortality and the number of organ-support-free days compared with usual care. However, the PRNT50 titer of CCP transfused in the CONCOR-1, RECAP-MAP study was only 160 IQR (80, 320) and ≥ 1:160, respectively, lower than our study. [22, 23].

Even though the data from the RECOVERY trial showed 1:300 titer of CPT could not show a discrepancy in mortality from the standard of care. 29.4% of CPT patients were hospitalized in the critical care unit on admission day. This might imply that more severe cases and prolonged symptoms before CCP transfusion (8 days) might have affected the mortality and clinical outcomes compared with the duration of symptoms before transfused CCP in our study (5.5 days) [11]. However, our study could not show that CPT reduced overall oxygen supplemental, including low or high-flow oxygen, invasive mechanical ventilation, or extra-corporeal membrane oxygenation at 28 days like a recent multicenter study [24].

However, a recent RCT failed to show a difference between CPT and usual care; low neutralizing antibodies of CCP might not be effective enough to demonstrate benefit in reducing mortality [9, 10]. In a study in patients with COVID-19-related symptoms for 10 days and classified as moderate, severe, and critical, the PRNT50 titer of CCP was 160 (IQR 80–640) and did not demonstrate an advantage in survival improvement, course of illnesses, or viral clearance [25]. The challenge of high-titer CPT early in the course of the disease remains an interesting issue.

A study in the Netherlands revealed that mortality with CPT with neutralizing antibody titer ≥ 1:80 was non-statistically significantly lower than usual care, which might have been because of the low-titer CPT [26].

Another study demonstrated that high-titer neutralizing antibody CPT demonstrated equivocal results in the rate of supplemental oxygen from baseline characteristics of severe COVID-19 patients, which did not correlate with very low supplemental oxygen requirement in the primary outcome [11]. A study in a respiratory care unit (RCU) revealed that CCP from donors with a high immunoglobulin G antibody titer level had more favorable outcomes than those with a low titer [27]. The study in severely ill patients transfused with mainly high neutralizing antibody titers of 1:640 within 3 days after COVID-19 was diagnosed led to successful weaning from oxygenation support after CPT within 3 days. Moreover, the radiologic improvement was demonstrated within 7 days. CPT within 14 days provides more favorable outcomes than after 14 days [28, 29].

Our study highlighted the benefit of higher neutralizing antibody level CPT in those with a shorter duration from onset of symptoms and earlier developed severe COVID-19 than other studies.

In our study, 15.3% of patients receiving CPT died from severe COVID-19 at 14 and 28-day, and one died from severe COVID-19 pneumonia with gastrointestinal bleeding and hyperglycemia. Almost all death cases from severe COVID-19 had delayed receiving CPT after 5 days of admission due to the physician waiting for RdRp inhibitor effect and had ≥ 2 comorbidities such as elderly, uncontrolled diabetes, and obesity. Other CPT studies have demonstrated that early CPT within 3 days after hypoxemia results in the greatest advantage.

According to many studies and meta-analyses, CPT might be an unsuitable treatment in late severe or critical COVID-19 owing to the pathophysiology that neutralizing antibodies might protect the SARS-CoV-2 from invading respiratory epithelial in early-stage of SARS-COV-2 infection [30].

The extended length of stay in the CCP receiving group (10 days) reflected the rapid deterioration, early death, and shortening stay in the standard of care group (7 days). The study in Mexico reported a prolonged stay of 22.5 days after CCP transfusion, in contrast to the 10 days in our study: the shorter stay might suggest the benefit of CPT in early developed severe COVID-19 [31]. Interestingly, although the invasive mechanical ventilation receiving rate was 85% in both groups, the successful weaning oxygenation support was 84.6% in the CPT group, much more than 14.5% in the control group. Moreover, Almost all CPT patients had clinical improvement within 7 days after CCP transfusion.

Our study supported the favorable CPT evidence in COVID-19, especially in those who earlier developed severe COVID-19. The CPT within 9 days after admission showed a benefit of 3.4% absolute risk reduction for individuals with risk factors for disease progression or vaccination status. The outpatient study demonstrated that early CPT reduced disease progression risk, leading to hospitalization [32]. Although the potential benefit of early high-titer CPT in mildly ill older adults demonstrated reduced deterioration to severe COVID-19, our data supported the benefit of CPT in a study in older adults and individuals with severe COVID-19 with or without comorbidities [33].

Although some CPT studies report cases of anaphylactic shock [28], our study demonstrated no serious adverse events, transfusion-related acute lung injury, or transfusion-associated circulatory overload. One patient developed a transfusion-related minor allergic reaction, which spontaneously recovered.

The limitation of our study is the real-world retrospective observational study in nature. The CPT and standard care groups were cared for by different groups of clinicians, albeit concurrently, during the Delta variant epidemic in Thailand. The lack of declared immunization status in both donor and recipient, previous SARS-CoV-2 infection, previous treatment or inadequate treatment data before admission, the patients' immunogenicity, or neutralizing antibodies testing to spike protein of SARS-CoV-2 in our study might influence the interpreting of the clinical outcomes from CPT. All patients who received RdRp inhibitor due to Thai standard treatment guidelines might affect the outcome. Resource limitations characteristic of a community hospital setting meant that essential biomarkers such as high-sensitivity C-reactive protein or ferritin to detect clinical deterioration were unavailable. However, hypoxemia in severe COVID-19 indicated deterioration and reflects worsening severity. The lack of virologic and immunologic studies is also a limitation of our study.

## Conclusions

The immediate high neutralizing antibodies titer of CPT transfused in earlier developed severe COVID-19 diagnosis was safe and related to decreasing death. Moreover, the CPT reduced the duration of supplemental oxygen and improved clinical and radiologic improvement. The factors that improved the clinical outcome in CPT included early CCP transfusion, short duration from onset of symptoms, and high neutralizing antibodies titer of CCP. Factors associated with clinical improvement with CPT included early CCP transfusion and a high neutralizing antibody titer. CPT may provide an option for the early treatment of newly developed severe COVID-19, especially in resource-limited countries where novel treatments are difficult to access. CPT might be hopeful agents to combat new VOCs. The CCP transfused in the early course of COVID-19 illness is very challenging and needs additional study.

## Data Availability

All data generated or analyzed during this study are included in this published article.
